# Unexpected Ipsilateral Pedicle Fracture in Nontraumatic Lumbar Spondylolysis: An Uncommon Case

**DOI:** 10.7759/cureus.105931

**Published:** 2026-03-26

**Authors:** Ryo Takahashi, Koji Hayashi, Kosuke Misaki, Katsunori Mizuno

**Affiliations:** 1 Department of Rehabilitation Medicine, Fukui General Hospital, Fukui, JPN; 2 Department of Orthopedics, Fukui General Hospital, Fukui, JPN

**Keywords:** : case report, ipsilateral, lumbar spondylolysis, mechanical stress, pedicle stress fracture

## Abstract

We describe a rare case of unexpected ipsilateral pedicle fracture in nontraumatic lumbar spondylolysis. A 54-year-old male fish market worker (height: 171 cm; weight: 91 kg; body mass index: 31.1 kg/m²) presented with a 30-day history of progressive low back pain following repetitive heavy lifting at work. His occupational duties involved repeatedly handling and loading approximately 20 kg boxes of fish onto trucks at a wholesale fish market. Physical examination revealed a visual analogue scale score of 50 mm, left lower extremity motor weakness in the L5 distribution (Medical Research Council (MRC) grade 4), and sensory disturbance in the same dermatome. The patient was a current smoker. Radiography and computed tomography demonstrated unilateral left-sided lumbar spondylolysis at the fifth lumbar vertebra (L5), accompanied by an ipsilateral pedicle fracture and low-grade spondylolisthesis at the same segment. Dual-energy X-ray absorptiometry demonstrated osteopenia (T-score -2.4 at L2-L4). Pedicle fractures associated with spondylolysis typically occur contralaterally as a compensatory biomechanical response; therefore, ipsilateral occurrence without trauma is exceedingly rare. In the present case, segmental instability related to spondylolisthesis may have altered posterior load transmission, potentially increasing repetitive mechanical stress on the ipsilateral pedicle. Conservative management failed, and the patient subsequently underwent transforaminal lumbar interbody fusion (TLIF) with pedicle screw fixation 115 days after initial presentation. Postoperatively, the low back pain and numbness resolved completely, while the motor weakness remained stable (MRC grade 4). He returned to work 130 days after initial presentation. This case contributes to the existing literature by demonstrating that the interaction of smoking, borderline low bone mineral density, elevated body mass index, repetitive occupational heavy lifting, and segmental instability may create a biomechanical environment that can produce atypical ipsilateral structural failure of the posterior spinal elements. In patients with unilateral spondylolysis and persistent neurological symptoms, clinicians should meticulously evaluate both pedicles rather than focusing solely on the contralateral side.

## Introduction

Spondylolysis is a stress fracture of the pars interarticularis frequently observed in young athletes and has a documented prevalence in adults [1]. A large-scale study in the Japanese population reported lumbar spondylolysis in 5.9% of individuals undergoing abdominal computed tomography, approximately 21% of which were unilateral [2]. Although unilateral spondylolysis often follows a relatively benign natural history [3], it may alter mechanical load distribution across the posterior elements and predispose adjacent structures to stress-related injury.

Pedicle stress fractures are uncommon because of the pedicle’s substantial cortical thickness and short moment arm, which confer intrinsic structural strength [4]. When pedicle fractures occur in association with unilateral spondylolysis, they are typically observed on the contralateral side as a compensatory biomechanical phenomenon resulting from load redistribution across the neural arch [5,6]. Indeed, several cases of unilateral spondylolysis associated with contralateral pedicle fractures have been reported [7,8,9].

Spondylolisthesis at the affected segment may further exacerbate instability and modify load transmission, increasing shear forces across the posterior elements. However, concurrent ipsilateral spondylolysis and pedicle fracture in the absence of traumatic insult remains exceedingly rare, and the biomechanical mechanisms underlying this atypical presentation have not been well characterized in the literature.

This report describes an unusual case of unilateral L5 spondylolysis accompanied by an ipsilateral pedicle fracture and low-grade spondylolisthesis in a middle-aged labor-intensive worker without traumatic injury. The patient was employed at a wholesale fish market, where his work involved repetitive lifting and loading of heavy fish containers. This case highlights a potential biomechanical interaction between occupational heavy lifting, elevated body mass index, smoking, borderline bone mineral density, and segmental instability that may predispose the posterior spinal elements to atypical structural failure.

## Case presentation

A 54-year-old male worker at a wholesale fish market presented with a 30-day history of progressive low back pain that developed during occupational activity. His work primarily involved repetitive lifting and loading of fish containers weighing approximately 20 kg onto transport trucks. These tasks required repetitive forward bending and substantial axial loading of the lumbar spine. The pain gradually intensified and interfered with his work activities. On initial evaluation, the visual analogue scale (VAS) score (0 mm: no pain; 100 mm: worst imaginable pain) for low back pain was 50 mm. The patient had no prior history of lumbar spine disorders or significant traumatic injury. He was a current smoker (10 cigarettes/day for 30 years) but had no known family history of spinal disease or metabolic bone disorders.

**Figure 1:**
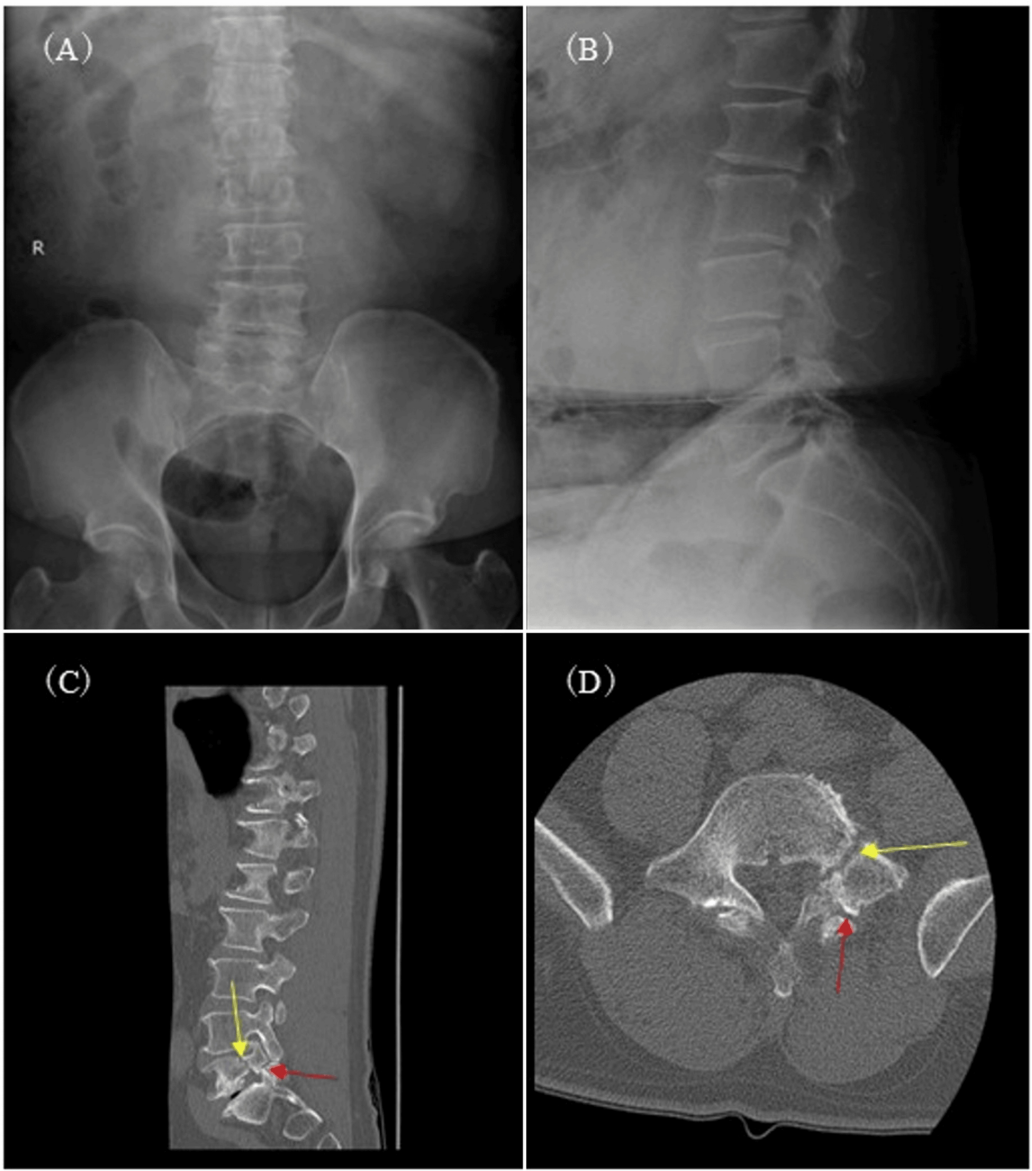
Lumbar X-ray and CT results CT: computed tomography Preoperative radiographs and CT images demonstrating L5 spondylolysis with an ipsilateral pedicle fracture. Left-sided spondylolysis (red arrows) and a concomitant ipsilateral pedicle fracture (yellow arrows) are clearly identified at the L5 level. (A) Anteroposterior radiograph, (B) lateral radiograph, (C) sagittal CT image, (D) axial CT image

**Figure 2:**
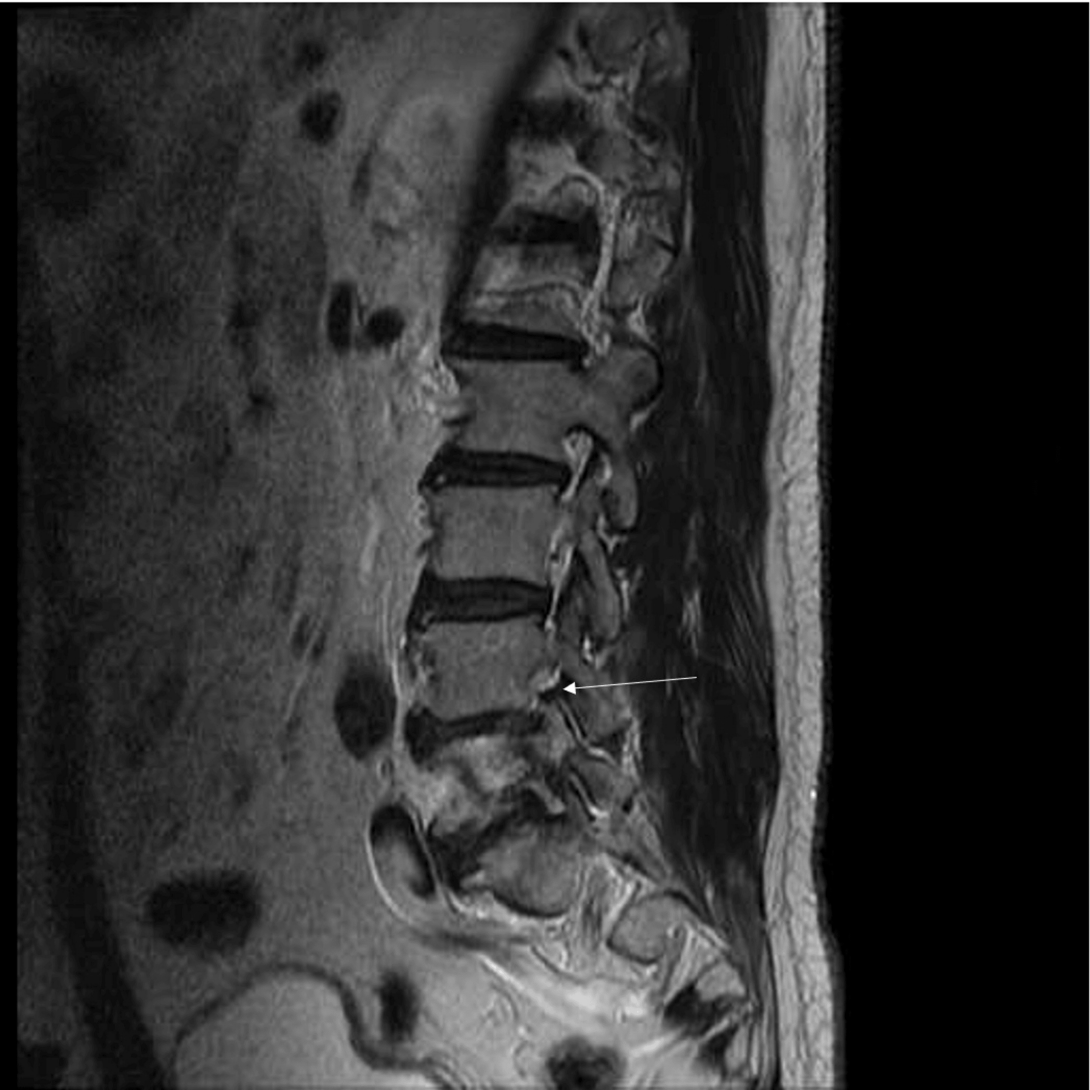
Preoperative lumbar MRI results on sagittal T2-weighted images MRI: magnetic resonance imaging Lumbar MRI demonstrates significant left-sided L5 foraminal stenosis (white arrow)

Neurological examination revealed left-sided motor weakness predominantly in the L5 distribution and numbness in the same dermatome. Manual muscle testing demonstrated tibialis anterior strength of Medical Research Council (MRC) grade 5 on the right and grade 4 on the left, and extensor hallucis longus strength of MRC grade 5 on the right and grade 4 on the left, while gastrocnemius-soleus complex strength was MRC grade 5 bilaterally. Deep tendon reflexes, including the patellar tendon reflex and Achilles tendon reflex, were normoactive bilaterally. The straight leg raise test was negative bilaterally at 75 degrees. Lumbar X-ray and computed tomography (CT) revealed unilateral left-sided lumbar spondylolysis at the L5 level with a concomitant ipsilateral pedicle fracture and associated low-grade spondylolisthesis at the same segment (Figures 1A-1D). CT clearly demonstrated cortical discontinuity of both the pars interarticularis and the pedicle (Figures 1C-1D). Dual-energy X-ray absorptiometry demonstrated a T-score of −2.4, consistent with osteopenia. Magnetic resonance imaging (MRI) demonstrated left-sided L5 foraminal stenosis, likely exacerbated by the structural compromise and associated spondylolisthesis (Figure 2). No evidence of lumbar disc herniation or significant bone marrow edema around the pedicle fracture was observed. Laboratory examination demonstrated no evidence of systemic inflammation (white blood cell count: 6300 /µL, reference range: 3300-8600 /µL; C-reactive protein: 0.05 mg/dL, reference range: 0.00-0.14 mg/dL), thereby excluding infectious spondylitis or neoplastic disease.

**Figure 3:**
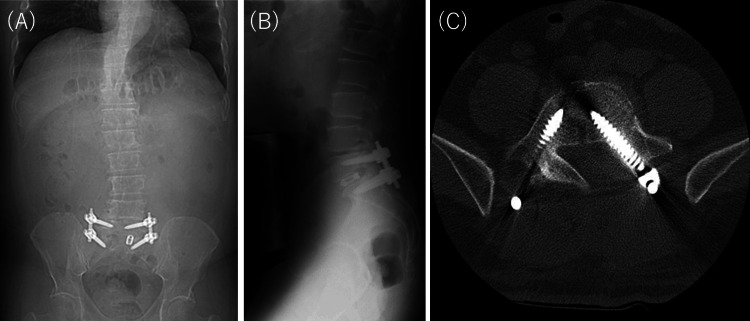
Postoperative lumbar X-rays and CT images CT: computed tomography; TLIF: transforaminal lumbar interbody fusion Postoperative lumbar X-rays and CT images following pedicle screw fixation and TLIF. (A) Anteroposterior radiograph, (B) lateral radiograph, (C) axial CT image

The patient initially underwent conservative management consisting of activity modification, oral medication including mirogabalin (5 mg/day), celecoxib (100 mg/day), and rebamipide (100 mg/day), use of a soft lumbar brace, and structured rehabilitation therapy. Conservative treatment was continued for approximately three months. During this period, low back pain, left-sided numbness, and motor weakness in the L5 distribution persisted without significant improvement. Follow-up imaging suggested persistent structural instability at L5. These findings indicated that the L5 nerve root was likely being compromised by dynamic mechanical instability within the stenotic foramen, resulting in failure of nonoperative treatment. The patient subsequently underwent transforaminal lumbar interbody fusion (TLIF) with pedicle screw fixation 115 days after initial presentation. Intraoperative findings confirmed instability at the affected segment. Postoperative imaging confirmed appropriate implant positioning and restoration of segmental alignment (Figure 3). The low back pain and numbness resolved completely following surgery. Although the motor weakness in the L5 distribution remained at MRC grade 4 without further improvement, the patient was able to return to work 130 days after initial presentation due to the relief of pain.

## Discussion

Unilateral lumbar spondylolysis alters the distribution of mechanical stress across the posterior elements and has been reported to predispose the contralateral pars interarticularis or pedicle to stress-related injury through compensatory load redistribution [5,6]. In contrast, pedicle fractures occurring on the same side as the pars defect are uncommon because the pedicle typically possesses substantial structural strength due to its thick cortical architecture and short lever arm [4].

In the present case, unilateral spondylolysis was accompanied by low-grade spondylolisthesis at the same segment. Vertebral slip may increase anterior shear forces and modify posterior load transmission [10], potentially concentrating repetitive mechanical stress on the ipsilateral pedicle-facet complex. This altered biomechanical environment may partially explain the unusual ipsilateral fracture pattern observed in this patient. In addition, foraminal stenosis at L5 was identified without a disc herniation, suggesting that neural symptoms were primarily related to structural compromise and segmental instability rather than soft-tissue pathology [11]. The patient exhibited both motor weakness and sensory disturbance in the L5 distribution preoperatively, which likely reflected dynamic mechanical irritation of the L5 nerve root within the narrowed foramen.

Although sagittal CT demonstrated a relatively sharp cortical margin along the pedicle fracture line, subtle adjacent sclerotic changes on axial images raise the possibility that the lesion represented progression of a pre-existing incomplete stress injury. This finding suggests that the fracture may represent part of a stress-fracture continuum rather than a purely acute structural failure. However, the precise temporal sequence cannot be determined without longitudinal imaging or metabolic evaluation.

Bone mineral density may also have contributed to the structural vulnerability observed in this case. Although the patient did not meet the formal diagnostic criteria for osteoporosis, the T-score of -2.4 indicates borderline reduction in bone mineral density and may reflect reduced bone strength [12]. When combined with smoking history, elevated body mass index, and repetitive occupational loading associated with frequent lifting of heavy containers, this borderline skeletal fragility may have increased susceptibility to stress-related structural failure of the pedicle. Repeated axial loading during heavy lifting may increase compressive and shear stresses across the posterior spinal elements, particularly in the presence of pre-existing unilateral spondylolysis and segmental slip. Nevertheless, given the single-case design, these biomechanical interpretations remain speculative.

From a therapeutic perspective, TLIF was selected rather than direct pars repair or isolated decompression because of the concomitant pedicle fracture, associated spondylolisthesis, and intraoperatively confirmed segmental instability. Direct repair alone would not adequately address anterior shear forces in the presence of pedicle compromise and vertebral slip. Furthermore, TLIF enabled direct decompression of the stenotic foramen and rigid stabilization of the motion segment [13].

Following surgical stabilization, the patient experienced complete resolution of low back pain and radicular sensory symptoms. Although the motor weakness in the L5 distribution persisted at MRC grade 4, this incomplete recovery may be attributable to chronic nerve root compromise or irreversible neural changes resulting from the 115-day interval between symptom onset and surgical intervention. Nevertheless, rigid stabilization provided by TLIF eliminated dynamic neural irritation, leading to substantial symptomatic relief and enabling the patient to return to his physically demanding occupation. This case highlights the importance of carefully evaluating the pedicle in patients with unilateral spondylolysis and persistent radicular symptoms, as atypical ipsilateral structural failure may occur under certain biomechanical conditions.

## Conclusions

Pedicle fractures associated with lumbar spondylolysis typically occur contralaterally as a compensatory response. This case reports an exceedingly rare presentation of concurrent ipsilateral pedicle fracture and segmental spondylolisthesis in the absence of acute trauma. The interaction of smoking, borderline bone mineral density, elevated body mass index, and occupational mechanical loading likely created a unique biomechanical environment predisposing the posterior elements to atypical structural failure. In patients with unilateral spondylolysis and persistent radicular symptoms, clinicians should maintain a high index of suspicion for atypical pedicle involvement rather than focusing solely on the contralateral side. When conservative management fails, surgical stabilization via TLIF can effectively resolve pain and sensory deficits, facilitating a successful return to labor-intensive occupations even if minor motor weakness persists.
